# Complete genome of *Xanthomonas citri* pv. *anacardii* strain CCRMTAQ13 (causal agent of angular leaf spot in cashew) from Brazil using long-read Nanopore technology

**DOI:** 10.1128/mra.00877-24

**Published:** 2024-11-26

**Authors:** Hiago Antonio Oliveira da Silva, Lucas Magalhaes de Abreu, Marco Aurelio Siqueira da Gama, Jose Huguet-Tapia, Peiqi Zhang, Victor Hugo Buttros, Jain Mukesh, Frank White, Samuel J. Martins

**Affiliations:** 1 Department of Plant Pathology, University of Florida, Gainesville, Florida, USA; 2 Department of Plant Pathology, Universidade Federal de Viçosa, Viçosa, Minas Gerais, Brazil; 3 Department of Agronomy, Universidade Federal Rural de Pernambuco, Recife, Pernambuco, Brazil; 4 Department of Biology, Universidade Federal de Lavras, Lavras, Minas Gerais, Brazil; University of Strathclyde, Glasgow, United Kingdom

**Keywords:** bacteria, gene, genome analysis

## Abstract

Here, we present a single contiguous genome sequence of *Xanthomonas citri* pv. *anacardii* (*Xca*; strain CCRMTAQ13), the causal agent of angular leaf spot in cashew, an important commodity crop in Brazil. Oxford Nanopore Sequencing Technology was used to assemble the genome of the *Xca* in a single contig of 5,086,757 bp with a 64.53% GC content. This genome sequence will provide a useful resource for studies on virulence mechanisms, including the identification of a single transcription activator-like effector gene of 3,359 bp.

## ANNOUNCEMENT

Cashew (*Anacardium occidentale*), is a tropical fruit tree from the *Anacardiaceae* family, native to South America mainly cultivated in the northeastern Brazil (1–3). The Cashew angular leaf spot disease is caused by pigmented and non-pigmented strains of *Xanthomonas citri* pv. *anacardii* (*Xca*) (4, 5). Here, the complete, single contiguous genome sequence of *Xanthomonas citri* pv. *anacardii* (CCRMTAQ13) using Oxford Nanopore technology (ONT) is presented, achieving a significantly improved assembly over a previously announced assembly (accession no. PESH00000000) (4).

Strain CCRMTAQ13 was isolated in 2009 from cash ew leaves in the state of São Paulo, municipality of Taquaritinga (21°24′23 ″S, 48°30′20 ″W), Brazil and provided by the culture collection of the Laboratory of Plant Bacteriology of the Universidade Federal Rural de Pernambuco and stored in the collection of Martins’ Lab at University of Florida. The strain CCRMTAQ13 was cultured in Luria-Bertani (LB) broth (6) at 28°C for 48 h under aerobic conditions, and the cells were harvested for DNA extraction using the Miniprep Kit (Zymo Research, Irvine, CA, USA). The purity and quantity of DNA were assessed using a NanoDrop One/One C microvolume UV-Vis spectrophotometer and Qubit 4 Fluorometer (Thermo Fisher Scientific, Waltham, MA, USA). The genome was sequenced using ONT. About 1 μg genomic DNA was used to prepare the sequencing library using the ligation sequencing kit (SQK-LSK109; Oxford, UK) following the instructions of the manufacturer. No fragmentation or size selection was conducted. The library was sequenced on an R9.4.1 flow cell using a MinION device (Oxford Nanopore Technologies) for 36 h.

Base calling was completed using Guppy version 4.4.1 (7) using a high accuracy model dna_r9.4.1_450bps_hac.cfg. The quality of the reads was evaluated with NanoPlot version 1.30.1 (8). A total of 3.1 GB data were generated, with 300× depth coverage, with a total number of reads of 101,574.0 and a read length N50 of 15,231.0. Sequencing artifacts, including adapter sequences, were removed using Porechop v.0.2.4 (7). Filtlong v.0.2.0 (9) was employed for read-quality and length filtering, setting a mean quality score threshold of 8 and a minimum length of 1,000 base pairs. The processed reads were assembled using Flye version 2.9 (10). The raw assembled reads obtained from Flye were polished with Medaka version 1.7.0 (11). Circlator v.1.5.5 was used to circularize the genome and fix the start point as the dnaA gene (12). The completeness of the assembly was assessed using Benchmarking Universal Single-Copy Orthologs (BUSCO v.5.4.3) using the Xanthomonadales database (13) and annotation was performed with PGAP v.6.8 (14).

**Fig 1:**
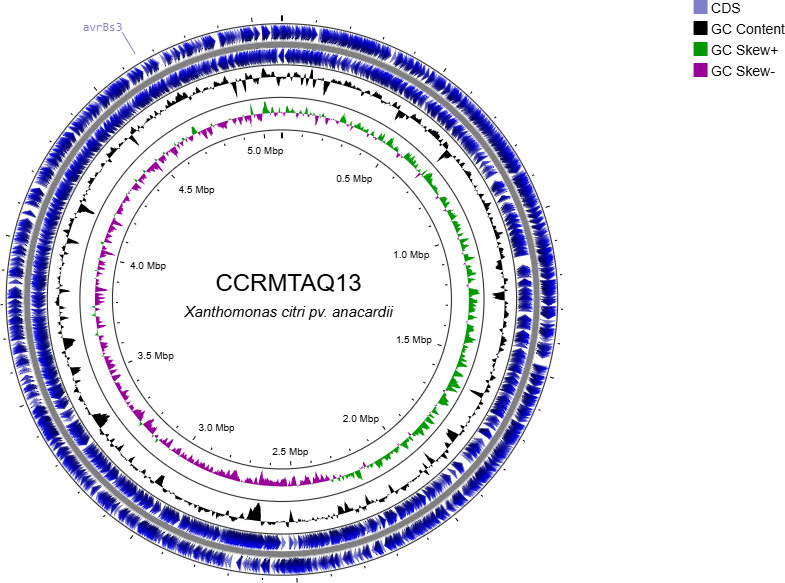
Genome of *Xanthomonas citri* pv. *anacardii* strain CCRMTAQ13. Rings (outermost to innermost) illustrate coordinates (kb), protein-coding genes on the forward (outer) and reverse strands, TALE gene (indicator), percent GC content, and GC skew. GC skew illustrates (G – C)/(G + C).

The final assembly produced a single circularized chromosome of 5,086,757 bp (same N50 number) with a GC content of 64.53% and no plasmids (Fig. 1). BUSCO analysis indicated 99.7% completeness, no duplicated or missing genes, and one fragmented gene (0.3%).

Annotation resulted in a better assembly with 4,247 coding genes, including a complete TALE gene (15). The TALE gene was fragmented in the previously available assembly (accession SAMN29417865), having only 1,003 nucleotides (Table 1).

**TABLE 1:** Genome annotation features of *Xanthomonas citri* pv. *anacardii* strain CCRMTAQ13

Sequencing technology	Nanopore*^a^*	Illumina*^b^*
Coding sequences	4,247	4,399
rRNA	6	7
tRNA	53	58
Contigs	1	231
Chromosomes	1	-
TALE genes	1	1
TALE size	3,359 nt	1,003 nt

^*a*^ Genome assembly statistics from 2023.

^*b*^ Previously short-read assembly from 2018 of CCRMTAQ13.

## Data Availability

This whole-genome project has been deposited in GenBank under the accession no. CP156748 and is also available online at https://github.com/Martins-Lab/Xanthomonas_citri_pv._anacardii_genome. The BioProject and BioSample for this project are PRJNA854066 and SAMN41265889, respectively. The SRA data is also deposited in GenBank under the accession no. SRR30110631. The version described in this paper is the second version of the short-read assembled genome accession no. PESH00000000.1 under project PRJNA416789.
